# Psychometric properties of the Symptom Checklist-90 in adolescent psychiatric inpatients and age- and gender-matched community youth

**DOI:** 10.1186/s13034-016-0111-x

**Published:** 2016-07-15

**Authors:** Minna Rytilä-Manninen, Sari Fröjd, Henna Haravuori, Nina Lindberg, Mauri Marttunen, Kirsi Kettunen, Sebastian Therman

**Affiliations:** Hospital District of Helsinki and Uusimaa, Kellokoski Hospital, 04500 Kellokoski, Finland; Adolescent Psychiatry, University of Helsinki and Helsinki University Hospital, Helsinki, Finland; School of Health Sciences, University of Tampere, Tampere, Finland; Department of Health, Mental Health Unit, National Institute for Health and Welfare, Helsinki, Finland; Forensic Psychiatry, University of Helsinki and Helsinki University Hospital, Helsinki, Finland

**Keywords:** Adolescent, Bifactor, Clinical, Differential item functioning, Factor structure, Measurement invariance, Psychometric property, Symptom Checklist-90, SCL-90, Validity

## Abstract

**Background:**

The Symptom Checklist-90 (SCL-90) is a questionnaire that is widely used to measure subjective psychopathology. In this study we investigated the psychometric properties of the SCL-90 among adolescent inpatients and community youth matched on age and gender.

**Methods:**

The final SCL-90 respondents comprised three subsets: 201 inpatients at admission, of whom 152 also completed the instrument at discharge, and 197 controls. The mean age at baseline was 15.0 years (*SD* 1.2), and 73 % were female. Differential SCL-90 item functioning between the three subsets was assessed with an iterative algorithm, and the presence of multidimensionality was assessed with a number of methods. Confirmatory factor analyses for ordinal items compared three latent factor models: one dimension, nine correlated dimensions, and a one-plus-nine bifactor model. Sensitivity to change was assessed with the bifactor model’s general factor scores at admission and discharge. The accuracy of this factor in detecting the need for treatment used, as a gold standard, psychiatric diagnoses based on clinical records and the Schedule for Affective Disorders and Schizophrenia for School-Age Children—Present and Lifetime (K-SADS-PL) interview.

**Results:**

Item measurement properties were largely invariant across subsets under the unidimensional model, with standardized factor scores at admission being 0.04 higher than at discharge and 0.06 higher than those of controls. Determination of the empirical number of factors was inconclusive, reflecting a strong main factor and some multidimensionality. The unidimensional factor model had very good fit, but the bifactor model offered an overall improvement, though subfactors accounted for little item variance. The SCL-90s ability to identify those with and without a psychiatric disorder was good (AUC = 83 %, Glass’s Δ = 1.4, Cohen’s *d* = 1.1, diagnostic odds ratio 12.5). Scores were also fairly sensitive to change between admission and discharge (AUC 72 %, Cohen’s *d* = 0.8).

**Conclusions:**

The SCL-90 proved mostly unidimensional and showed sufficient item measurement invariance, and is thus a useful tool for screening overall psychopathology in adolescents. It is also applicable as an outcome measure for adolescent psychiatric patients. SCL-90 revealed significant gender differences in subjective psychopathology among both inpatients and community youth.

## Background

Adolescence is a transitional stage from childhood to adulthood during which the individual undergoes many physiological, psychological, cognitive, and social changes. It is a risk period for the emergence of many psychiatric disorders [1, 2]. The incidence of psychiatric disorders increases from childhood through mid-adolescence, peaking in late adolescence and young adulthood [3], and approximately one adolescent in five suffers from a psychiatric disorder [4]. In Finland, about 3 % of the adolescent population (ages 13–22) is referred to adolescent psychiatric secondary care, and approximately 0.4–0.6 ‰ require psychiatric hospitalization [5].

Symptom inventories provide an economical means of assessing adolescents’ mental disturbance levels and treatment effectiveness. As Symptom Checklists and rating scales provide extensive amounts of clinical information relatively quickly, self-report symptom inventories are commonly used by both clinicians and researchers to gather information on patients’ mental states. Furthermore, self-report questionnaires can be used to monitor the quality of medical and psychological interventions in mental health services, and to screen for symptoms of psychopathology [6]. Because psychiatric comorbidity is typical for adolescents with mental disorders, a growing body of research has supported using multidimensional scales [7]. One such questionnaire is the Symptom Checklist-90 (SCL-90) [8], a widely applied self-assessment tool for individuals with a broad range of mental disorders and symptom intensity. It contains 90 items and takes approximately 12–15 min to administer, yielding nine scores for primary symptom dimensions and three for global distress. The symptom dimensions comprise somatization, obsessive–compulsive behavior, interpersonal sensitivity, depression, anxiety, hostility, phobic anxiety, paranoid ideation, and psychoticism [8]. The main global index of distress is the global severity index (GSI), which is the average of all responses. A time reference of 1–2 weeks is usually used.

The SCL-90 has been tested in different settings, including community [6, 9–13] and psychiatric outpatient [14, 15] and inpatient samples [16–18]. It is commonly used as an indicator of change in symptoms [19, 20] and as a treatment outcome measure [21, 22]. The SCL-90s ability to discriminate patients from non-patients is adequate [13, 14], but correlations with analogous and non-analogous measures have been somewhat controversial [17, 23]. Significant gender differences have also emerged [13, 21, 24]. The main criticism of the instrument, however, has focused on the original 9-factor structure, with substantial difficulties arising in its replication. One general factor accounting for a large proportion of variance has been proposed in some studies with adults [14, 17, 19, 25].

The aim of the present study was to investigate the measurement invariance, factor structure, reliability, and validity of the SCL-90 among adolescents. A new approach is the use of a bifactor model, which according to Reise [26], is effective when modeling construct-relevant multidimensionality. A bifactor model consists of general factor and a number of specific factors, allowing each item to load both on the general factor and specific factor [26, 27]. In this study we compare two groups, inpatients and controls, and also the same patient sample at two time points, namely admission and discharge. As a prerequisite for comparing these two groups and two time points accurately, a measurement invariance analysis was executed. Measurements invariance signifies that the association between the items and the latent factors should not depend on group membership or measurement occasion, but the measurement instrument and the construct being measured are operating in the same way across diverse samples of interest [28].

To the best of our knowledge, this is the first study that examines the dimensionality and viability of the SCL-90 subscale scores in an adolescent sample by applying a bifactor model. In line with recent findings supporting a bifactor model of the SCL-90 with adults [29], we expect that the model with nine specific factors and one general factor of symptoms would be the best fitting solution. Our second aim is to estimate the screening performance of the SCL-90 and to determine optimal cut-off point. To our knowledge, there are no discrimination thresholds for distinguishing between adolescent patients and the general population or between adolescents with a diagnosed mental disorder and those without. An earlier study in a Finnish adult sample [10] has shown that the screening properties of this SCL-90 translation are good.

The findings could provide important information on the best practices for using the SCL-90 questionnaire and interpreting SCL-90 scores among adolescents.

## Methods

### Participants and procedure

#### Inpatients

The Kellokoski Hospital Adolescent Inpatient Follow-Up Study (KAIFUS) is a longitudinal naturalistic study on clinical characteristics and impact of treatment in a consecutive sample of adolescent psychiatric inpatients in Southern Finland. The sample comprises 13- to 17-year-old adolescents admitted to Kellokoski Hospital for the first time between September 2006 and August 2010 (*N* = 395). We excluded adolescents with a treatment period of less than 2 weeks, with intellectual disability, with an age under 13 years, or with a poor knowledge of Finnish language (*n* = 80, 20 %). Furthermore, 62 adolescents (16 %) declined to participate, 23 (6 %) discontinued their treatment, and 24 (6 %) had incomplete data. The final inpatient admission sample comprised 60 boys (29 %) and 146 girls (71 %) with a mean age of 15.1 years (*SD* = 1.2). Non-participation was unrelated to age (*p* = 0.31, two-sided t test), living situation (*p* = 0.58), socioeconomic status (*p* = 0.38), or the presence of substance use disorders (*p* = 0.59), mood disorders (*p* = 0.92), conduct disorder (*p* = 0.09), anxiety disorders (*p* = 0.39), or eating disorders (*p* = 0.34), but was higher among boys (*p* = 0.02) and among patients with psychotic disorders (*p* = 0.02). Patients were diagnostically interviewed with the Schedule for Affective Disorders and Schizophrenia for School-Age Children—Present and Lifetime version [30]. The patients were requested to complete the SCL-90 at the beginning of their stay as well as at discharge. The treatment duration was between 31 and 90 days in 38 % of the cases, 42 % of the patients stayed in hospital for over 90 days, and 20 % of the patients for less than 31 days. For more details, see Rytilä-Manninen et al. [31]. The study was designed to detect clinically meaningful group differences, and the planned sample size of 200 patients and 200 controls is sensitive enough to achieve 80 % power even for small effect sizes (*d* > 0.28) when α is set to 0.05 on a *t* test.

#### Community sample

The control group comprised a random sample of sex- and age-matched students from two secondary, one vocational, and four comprehensive schools, collected from the same geographical area as the inpatients. A total of 473 students were invited; 202 (43 %) refused to participate, and 68 (14 %) failed to complete the self-assessments despite providing consent. The final sample consisted of 55 males (27 %) and 148 females (73 %). All were native Finns, with a mean age of 14.9 years (*SD* = 1.2). No significant differences were found between adolescents who participated and those who did not with regard to socioeconomic status (*p* = 0.61) or living situation (*p* = 0.49). The same interviews and questionnaires were used with the community youth group as with patients. Based on the diagnostic interviews, 21 % of these youths met the criteria for at least one psychiatric disorder. For more details, see Rytilä-Manninen et al. [31].

#### Ethical aspects

Participation was voluntary, and all participants and their legal guardians were required to provide written informed consent after receiving both verbal and written information about the study. The Ethics Committee of Helsinki University Hospital approved the study protocol. Permission to conduct the study was granted by the authorities of the Helsinki and Uusimaa Hospital District and school administrations. The study was performed in accordance with the Declaration of Helsinki.

### Measures

#### Schedule for affective disorders and schizophrenia for school-age children—present and lifetime version (K-SADS-PL)

Psychiatric diagnoses were assessed based on the K-SADS-PL interview [30]. This is a semi-structured interview with good to excellent test–retest reliability and high concurrent validity and inter-rater agreement between the original and translated versions [30, 32–34]. The Finnish translation has previously been used in studies of both adolescent in- and outpatients [35, 36].

Psychiatrists specialized in treating adolescents assigned the psychiatric diagnoses according to the Axis-I disorders in DSM-IV [37] based on the K-SADS-PL and clinical records. Discrepancies were resolved by consensus between the psychiatrists. The psychiatric diagnoses present at the time of the baseline interview were included in the analyses, here dichotomized as having at least one psychiatric diagnosis present or no psychiatric diagnosis present.

#### Scl-90

SCL-90 is a self-report measure for persons aged at least 13 years. It consists of 90 items that represent nine factors and seven additional questions that are configure items, primarily concerning disturbances in appetite and sleep patterns, and are not scored collectively as a dimension [8]. Each of the nine symptom dimensions contains 6-13 items. Items are rated on a five-point Likert-scale of distress, ranging from “not at all” (0) to “extremely” (4). The General Severity Index (GSI) is the average score for all responded items and serves as an overall measure of psychiatric distress. In this study, the time of reference for the symptoms was the previous two weeks.

### Statistical analyses

#### Measurement invariance

To establish sufficient measurement invariance across groups and time points, an iterative algorithm was employed to detect differential item functioning (DIF) under Samejima’s graded response model for the full SCL-90, using the lordif package version 0.3–2 [38] for R with default settings (α = 0.01). The algorithm uses items tentatively flagged as invariant as anchors in an iterative process until a stable solution is identified. Patient responses at admission were separately compared with responses at discharge and control group responses. Total item-wise DIF was measured with summed uniform and non-uniform McFadden pseudo-*R*^2^.

#### Optimal number of factors

The multifactoriality of the subsample datasets were investigated with a number of indices for the optimal number of factors to extract: very simple structure (VSS), minimum average partial correlation (MAP), and parallel analysis (PA) [39–41]. These were calculated with the psych package version 1.5.8 in R version 3.2.3, using the polychoric correlation matrix and both weighted least-squares (WLS) and maximum likelihood (ML) estimation. VSS was investigated at complexity one and two, where an item is allowed to load on one or two factors only. In addition, the comparison data approach of Ruscio and Roche [42] was used, as implemented in R code supplied by the authors, using Spearman correlation matrices derived from complete cases.

#### Factor analyses

After establishing sufficient measurement invariance, the one-dimensional and a priori nine-dimensional model of the SCL-90 was fitted in confirmatory factor analyses (CFA) separately for patients at admission, patients at discharge, and controls.

In addition, in light of the evidence for a strong main factor, a bifactor model was specified with a general factor uncorrelated with the nine subfactors, which correlated with each other. The percentage of common variance attributable to the general factor was expressed with the explained common variance index (ECV) and the usefulness of individual subscales was assessed with McDonald’s omega hierarchical ω_h_ and omega subscale ω_s_ [26].

All factor analyses used the weighted least squares mean and variance adjusted (WLSMV) algorithm for categorical indicators in Mplus 7.3 [43], which performs well with skewed ordinal variables [44, 45] and with smaller samples [46]. Three fit indices were employed; for the comparative fit index (CFI) and the root mean square error of approximation (RMSEA) we followed the suggested cut-off values of Hu and Bentler [47] in judging adequacy of fit: >0.95 for CFI and <0.06 for RMSEA; for the weighted root mean square residual (WRMR) Yu [48] has suggested a cut-off of <1.0 under non-normality and small samples. Note that the one-dimensional and bifactor models included the six items not assigned to any of the nine subfactors. *Maximum* a posteriori factor scores were calculated for the bifactor model general factor.

#### Criterion validation

The three response sets of patients at admission, patients at discharge, and controls were compared on their SCL-90 general factor scores. As score distributions were approximately normal, Welch’s unequal variances *t*-test was employed (two-tailed, α = 0.05), and effect sizes were expressed with Glass’s Δ (using control/healthy variance only) and Cohen’s *d* (pooled variance). Similarly, diagnosed individuals were compared with non-diagnosed individuals in the combined admission and control groups. Gender effects were examined in all three response sets. Receiver operating characteristic (ROC) curves and associated area under the curve (AUC) values with non-parametric confidence intervals were computed with the pROC package [49] version 1.1-2 in R. The optimal cut-off point for discriminating between groups was determined with Youden’s *J* statistic [50], maximizing the sum of sensitivity and specificity. The overall discriminability at the chosen cut-offs was expressed as diagnostic odds ratios (DOR).

## Results

### Basic item distribution properties of SCL-90

From admission, discharge, and control sets 0.1, 0.4 and 0.2 % of SCL-90 responses were missing, respectively, with no individual having more than 30 missing responses. All models and scores were therefore estimated using all available data, assuming missingness at random. There was a strong floor effect in response distributions (item-wise skewness averaged 0.7 at admission, 1.6 at discharge, and 2.0 for controls), which in combination with the five-point response scale confirmed the necessity of employing factor analyses suitable for ordered categorical indicators.

### Measurement invariance

**Fig. 1:**
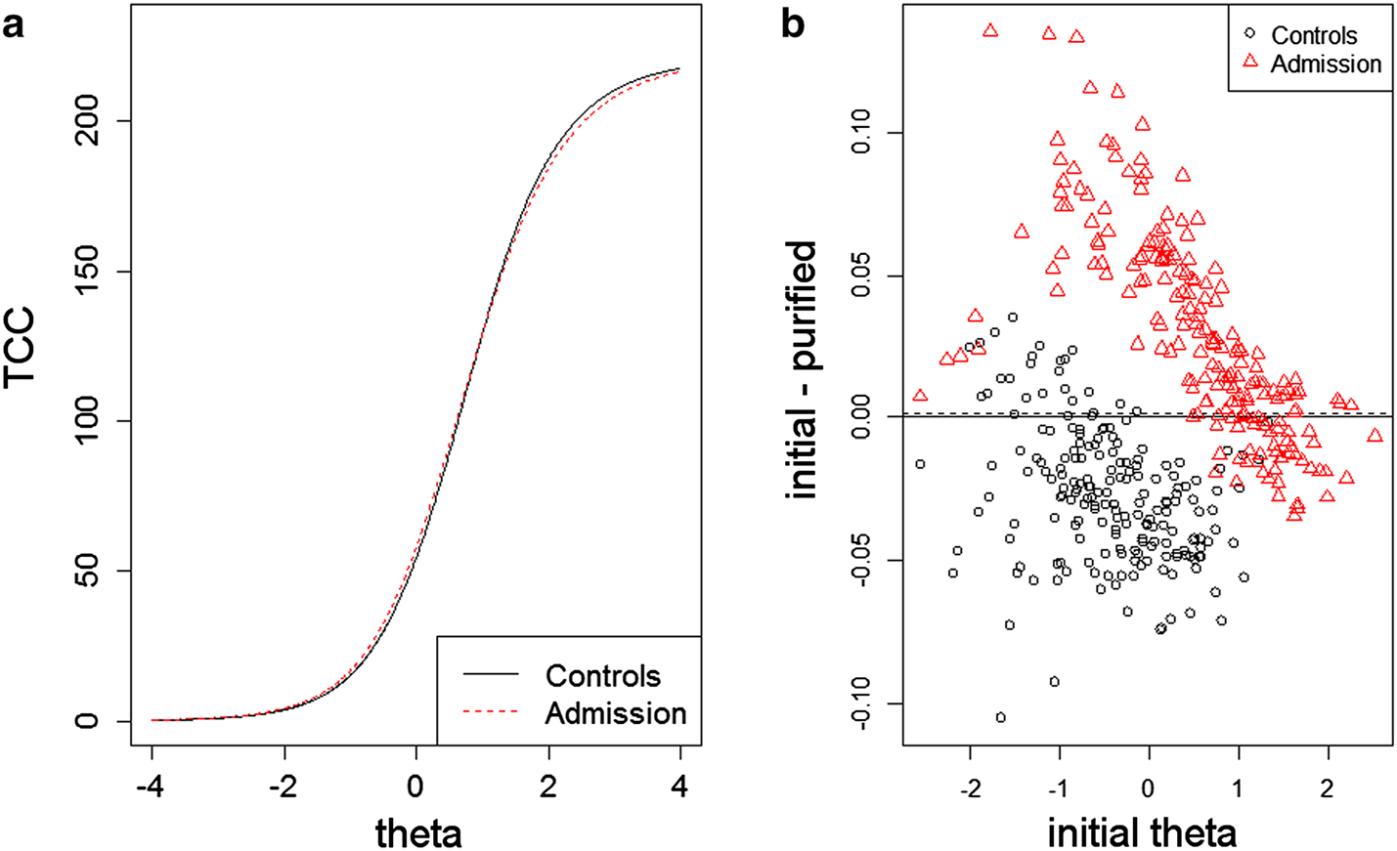
**a** Test characteristic curves by group based on all items. **b** Group-wise impact on theta estimates from accounting for DIF

When investigating the measurement invariance of items between patients and controls in the one-dimensional model, the iterative algorithm converged after 4 rounds, flagging 23 items for DIF, and McFadden *R*^2^ values for all items had a mean of 0.8 % and a median of 0.4 %. The highest values were observed for items 15 and 22 at 5.2 and 5.1 %. However, the total effect of the DIF of all items was small, as it was estimated to lead to 0.06 higher normalized latent scores in the patient group. Group-wise test characteristic curves and the impact of DIF are presented in Fig. 1.

When comparing admission and discharge responses of patients, the algorithm also converged after four rounds, flagging 11 items. McFadden *R*^2^ values for all items had a mean of 0.5 % and a median of 0.3 %, the highest values being 2.6, 2.5 and 2.3 % for items 32, 15, and 59, respectively. Again, the total effect of DIF was minimal, resulting in 0.04 higher scores at admission.

### Optimal number of factors

The empirical number of factors using WLS and ML estimation were almost identical, and only the former results are shown, along with results for the comparison data method, in Table 1. The various indices were highly divergent, with nominated number of factors ranging from one to nine, consistent with a complex factor structure with a strong primary factor.

**Table 1:** Suggested number of factors by various indices

Subsample	VSS complexity 2	MAP	Empirical BIC	PA	CD
Admission	2	5	5	9	1
Discharge	2	9	2	2	1
Controls	2	7	5	7	1

*VSS* very simple structure; *MAP* minimum average partial; *BIC* Bayesian information criterion; *PA* parallel analysis; *CD* comparison data method

### Confirmatory factor analyses

**Fig. 2:**
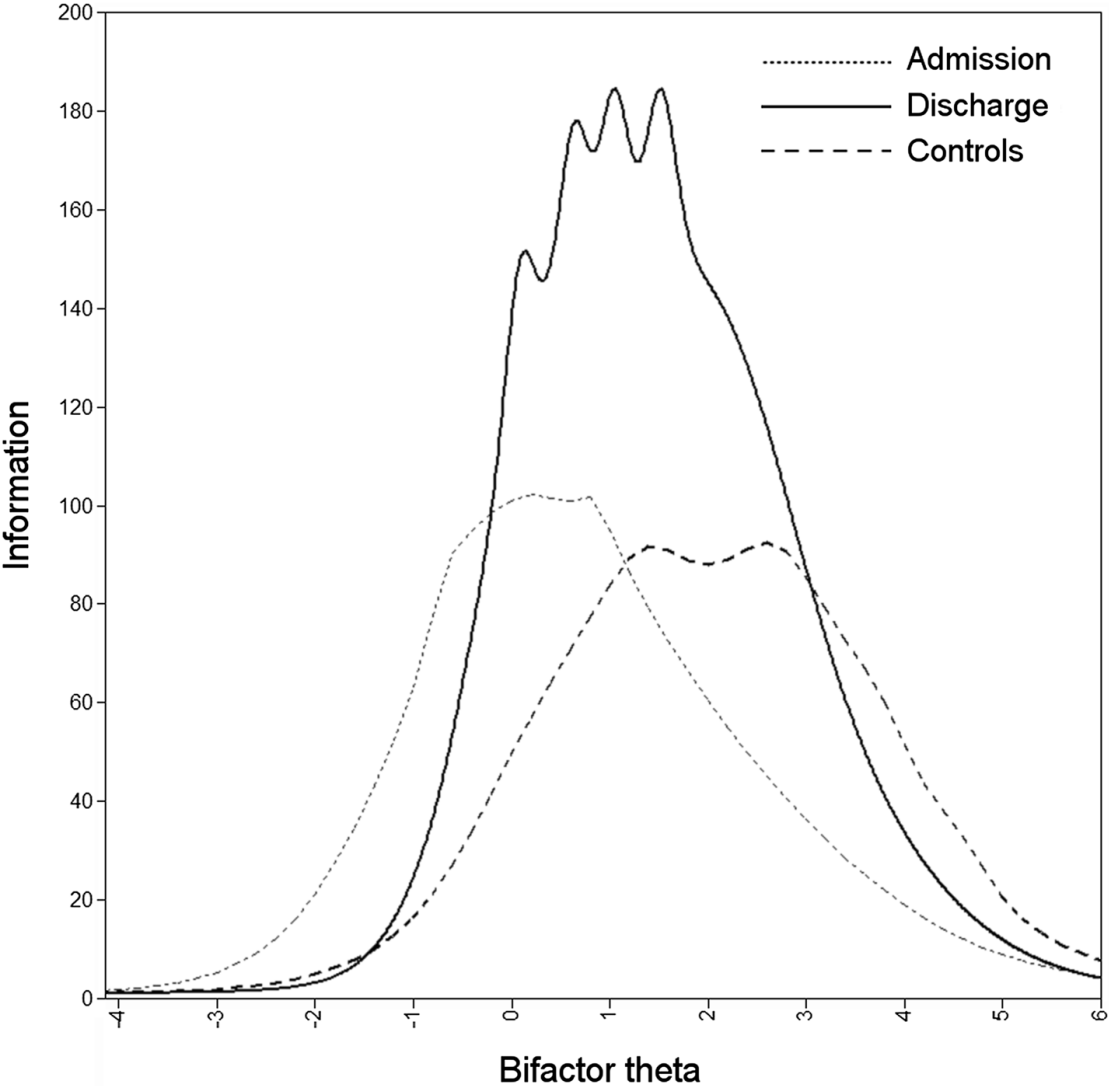
Total information curves as a function of theta for the general factor in admission (*dotted line*), discharge (*solid line*), and control subsamples. Note that the theta scale is normalized separately in each subsample

The one-dimensional CFA models had good fit in all three subsamples (Table 2). In contrast, the fit was poor for the a priori nine-dimensional models, and latent factors were very strongly correlated; the median inter-factor correlations were 0.84, 0.88, and 0.86 for the admission, discharge, and control datasets, respectively. The bifactor models had an even better fit than the corresponding one-dimensional models in the same subsamples. However, successfully fitting the bifactor models required leaving out item 15 from the depression subfactor, as the item was almost perfectly correlated with the general factor. Fit statistics of all models are presented in Table 2, and factor loadings, thresholds, and subfactor correlations of the patient admission subsample in Table 3. Total information curves of the general factor in the three subsamples are presented in Fig. 2.

**Table 2:** Fit statistics for CFA models

Dimensions	Items	Subsample	CFI	RMSEA	WRMR	Explained variance (%)
1	90	Admission	0.94	0.05	1.32	48
		Discharge	0.97	0.04	1.14	57
		Controls	0.96	0.03	1.10	43
9	84	Admission	0.50	0.13	3.53	56
		Discharge	0.66	0.13	3.43	64
		Controls	0.76	0.07	2.18	50
1 + 9 (bifactor)	90	Admission	0.97	0.03	1.02	58
		Discharge	0.98	0.03	0.93	66
		Controls	0.98	0.02	0.94	51

*CFI* comparative fit index; *RMSEA* root mean square error of approximation; *WRMR* weighted root mean square residual

**Table 3:** Standardized thresholds and factor loadings of nine-dimensional bifactor model of patient admission responses to SCL-90

Subfactor	Item	Thresholds				Explained variance (%)	Loadings	
		1	2	3	4		General factor	Subfactor
Somatization	48	0.06	0.63	1.26	1.81	71	0.48	0.70
	49	0.08	0.69	1.31	1.81	61	0.48	0.61
	56	0.05	0.76	1.20	1.70	68	0.56	0.61
	52	−0.09	0.54	1.08	1.65	52	0.40	0.60
	27	0.35	0.81	1.41	1.96	41	0.33	0.55
	58	0.31	0.92	1.52	2.33	50	0.48	0.52
	12	0.32	0.90	1.44	2.06	35	0.31	0.51
	42	−0.07	0.65	1.38	1.88	43	0.42	0.51
	4	−0.66	0.19	0.82	1.51	46	0.48	0.49
	53	0.04	0.65	1.31	1.88	51	0.54	0.47
	40	−0.32	0.34	1.11	2.06	41	0.47	0.44
	1	−0.88	−0.02	0.86	1.56	26	0.44	0.26
Obsessive–compulsive	65	0.29	0.77	1.15	1.44	58	0.34	0.68
	51	−0.51	0.13	0.71	1.41	64	0.52	0.61
	9	−0.69	−0.02	0.63	1.26	55	0.44	0.59
	45	−0.22	0.50	0.98	1.65	62	0.54	0.58
	38	−0.20	0.54	1.13	1.96	66	0.60	0.55
	46	−0.79	−0.14	0.51	1.25	63	0.62	0.50
	55	−0.78	−0.16	0.28	1.00	57	0.61	0.45
	28	−0.65	−0.02	0.59	1.08	76	0.76	0.43
	10	−0.46	0.08	0.69	1.38	48	0.56	0.41
	3	−0.74	−0.30	0.20	0.85	55	0.65	0.36
Interpersonal sensitivity	73	−0.36	0.11	0.69	1.20	70	0.54	0.65
	69	−0.11	0.41	0.87	1.40	72	0.57	0.63
	61	−0.82	−0.10	0.45	1.02	70	0.62	0.56
	37	−0.55	0.06	0.74	1.25	63	0.64	0.47
	36	−0.57	0.07	0.56	1.18	61	0.63	0.46
	34	−0.36	0.32	0.92	1.44	50	0.56	0.44
	6	−0.21	0.53	1.26	1.81	32	0.42	0.38
	21	−0.09	0.48	1.20	1.70	23	0.32	0.36
	41	−0.71	−0.17	0.44	0.85	76	0.86	0.16
Depression	5	−0.08	0.45	1.02	1.59	53	0.53	0.50
	14	−0.88	−0.24	0.40	1.02	64	0.67	0.44
	31	−1.00	−0.47	0.17	0.88	67	0.70	0.42
	29	−0.71	−0.07	0.40	0.90	63	0.68	0.41
	71	−0.72	−0.20	0.35	0.95	76	0.78	0.39
	22	−0.62	−0.23	0.49	1.15	64	0.71	0.38
	32	−0.66	−0.06	0.54	1.06	54	0.64	0.36
	30	−1.04	−0.59	0.08	0.74	79	0.82	0.34
	54	−0.57	−0.07	0.51	1.04	71	0.78	0.32
	20	−0.66	−0.17	0.35	1.02	43	0.59	0.28
	79	−0.48	0.03	0.44	0.95	80	0.86	0.23
	26	−0.59	−0.02	0.49	1.02	68	0.80	0.21
	15	−0.39	0.02	0.39	0.92	88	0.94	0.00
Anxiety	39	0.06	0.51	1.18	1.70	58	0.43	0.63
	72	−0.13	0.47	1.06	1.44	73	0.58	0.62
	33	−0.38	0.11	0.69	1.26	71	0.60	0.59
	57	−0.52	0.04	0.72	1.48	71	0.62	0.57
	23	−0.02	0.56	1.04	1.52	63	0.56	0.57
	17	−0.04	0.57	1.13	1.60	56	0.53	0.53
	80	0.05	0.50	1.02	1.60	50	0.57	0.42
	2	−1.31	−0.66	−0.04	0.92	59	0.65	0.41
	78	−0.24	0.36	1.02	1.51	51	0.60	0.39
	86	−0.18	0.41	0.91	1.44	53	0.67	0.28
Hostility	67	0.24	0.92	1.31	1.81	79	0.49	0.74
	63	0.03	0.82	1.15	1.65	62	0.37	0.70
	81	−0.18	0.37	0.92	1.28	70	0.61	0.58
	24	−0.50	0.16	0.73	1.41	52	0.56	0.46
	11	−0.95	−0.29	0.39	1.06	54	0.58	0.45
	74	0.03	0.81	1.51	2.05	29	0.38	0.38
Phobic anxiety	47	0.43	0.88	1.15	1.60	69	0.25	0.79
	70	−0.33	0.29	0.76	1.20	72	0.41	0.74
	50	−0.06	0.38	0.88	1.20	80	0.52	0.73
	13	−0.18	0.31	0.71	1.26	79	0.51	0.73
	25	0.34	0.68	1.15	1.65	60	0.49	0.60
	82	0.45	0.97	1.51	1.96	43	0.46	0.47
	75	−0.09	0.41	0.99	1.65	45	0.56	0.37
Paranoid ideation	43	−0.49	0.02	0.54	1.00	70	0.57	0.61
	18	−0.59	−0.03	0.50	1.08	63	0.53	0.59
	68	−0.14	0.47	0.92	1.37	64	0.59	0.54
	83	−0.01	0.66	1.02	1.48	49	0.51	0.48
	8	0.04	0.74	1.23	1.96	18	0.08	0.42
	76	−0.10	0.69	1.23	1.65	44	0.54	0.39
Psychoticism	62	0.33	0.88	1.31	1.88	59	0.52	0.57
	87	0.10	0.69	1.04	1.51	60	0.55	0.55
	84	0.67	1.19	1.88	2.17	42	0.37	0.54
	16	0.57	1.02	1.38	1.70	44	0.41	0.52
	7	0.39	0.92	1.48	2.17	39	0.41	0.48
	35	−0.14	0.44	0.98	1.52	56	0.59	0.47
	90	−0.31	0.08	0.47	0.97	58	0.65	0.39
	88	0.14	0.67	1.18	1.44	45	0.57	0.36
	77	−0.30	0.16	0.64	1.22	64	0.75	0.26
	85	0.24	0.74	1.10	1.75	41	0.60	0.21
(None)	19	−0.42	0.13	0.76	1.28	35	0.59	–
	44	−0.66	−0.14	0.39	1.04	57	0.76	–
	59	−0.56	−0.11	0.27	0.74	94	0.97	–
	60	0.27	0.71	1.06	1.44	30	0.55	–
	64	−0.41	0.19	0.79	1.44	37	0.60	–
	66	−0.40	0.11	0.57	1.13	62	0.79	–
	89	−0.45	0.06	0.52	1.10	78	0.89	–

As sufficient measurement invariance was established, *maximum* a posteriori factor scores for the general factor were estimated for all groups using the parameters of the patient admission bifactor model, which was the most multi-factorial of the three and had the most stable parameter estimates; the two items (15 and 22) showing a total DIF effect of over 5 % in either analysis were left out. Factor scores were standardized to set the control sample mean to zero and standard deviation to one, and are presented in Table 4. In the combined admission and control sample, the Pearson correlation between the GSI and factor scores was 0.956 and the Spearman correlation was 0.997, indicating very strong agreement with a curvilinear relationship.

**Table 4:** Score distributions and group comparisons

Subsample	N	Raw item means			Standardized general factor		
		Mean (SD)		Range	Mean (SD)		Range
Patients at admission	201	1.28 (0.79)		[0, 3.19]	1.37 (1.36)		[−2.8, 4.3]
Patients at discharge	152	0.68 (0.64)		[0, 2.52]	0.26 (1.39)		[−2.8, 3.1]
Controls	197	0.49 (0.41)		[0, 1.86]	0 (1)		[−2.8, 2.2]

### Subscale viability

The ECV of the general factor in the bifactor analyses was 56 % for the admission sample, 76 % at discharge, and 82 % for controls. McDonald’s omega values for the general factor and subscales are shown in Table 5.

**Table 5:** Viability of subscales in bifactor models

Scale	Subsample		
	Admission	Discharge	Controls
Omega-hierarchical (ω_h_)	0.89	0.95	0.97
Omega-subscale (ω_s_)			
Somatization	0.28	0.22	0.12
Obsessive–compulsive	0.28	0.15	0.07
Interpersonal sensitivity	0.23	0.10	0.04
Depression	0.12	0.03	0.02
Anxiety	0.26	0.12	0.07
Hostility	0.32	0.25	0.25
Phobic anxiety	0.42	0.28	0.23
Paranoid ideation	0.28	0.15	0.13
Psychoticism	0.20	0.15	0.09

### Group differences

The GSI scores by group are shown in Table 4. Using the standardized general factor scores from the bifactor model, boys had lower scores than girls in both admission (Welch test *p* < 0.001, Cohen’s *d* = 0.8; girls *M* = 1.7, *SD* = 1.2; boys *M* = 0.6, *SD* = 1.4) and control samples (*p* < 0.001, *d* = 0.6; girls *M* = 0.1, *SD* = 1.0; boys *M* = −0.4, *SD* = 1.0).

**Fig. 3:**
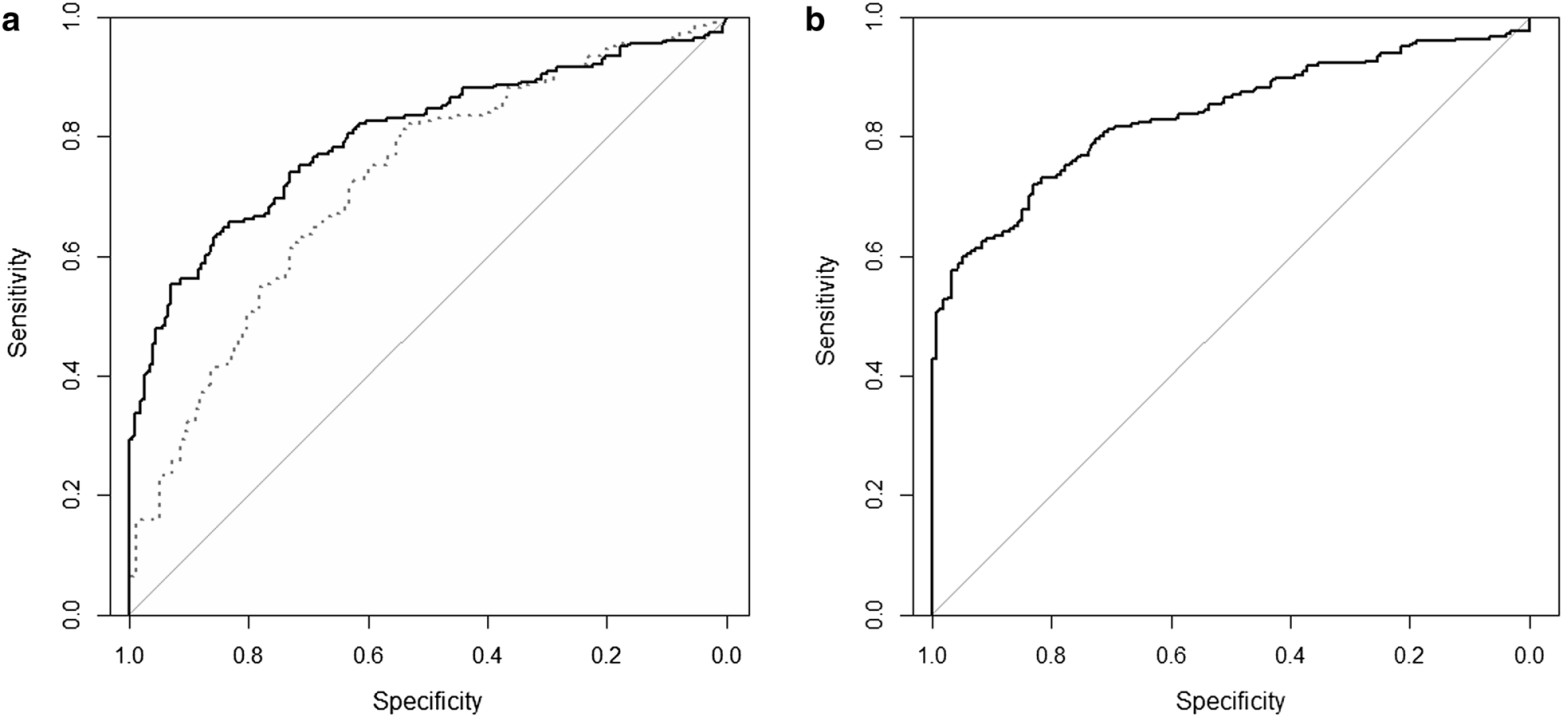
Receiver operating curves for the SCL-90 general latent factor score differentiating between (**a**) admission vs. discharge (*dotted line*) and admission vs. controls (*solid line*) and (**b**) individuals with or without a diagnosis in the combined admission and control sample

In the ROC analyses of the factor scores, adequate discrimination was found between patients at admission and discharge (AUC 72, 95 % CI [66.8, 77.4 %]) as well as between patients at admission and controls (AUC 79 % [75.5, 84.3 %]). Formulated differently, the group difference between patients at admission and controls was statistically highly significant and the effect was large (*p* < 0.001, Glass’s Δ = 1.4, Cohen’s *d* = 1.1). Patients’ scores were also significantly lower at discharge than at admission (paired test *p* < 0.001, *d* = 0.8). The optimal cut-off point to distinguish between controls and patients at admission was at θ = 1.14, approximately corresponding to a GSI of 0.99, providing 86 % specificity, 63 % sensitivity, and a DOR of 10.5. In the combined admission and control sample, individuals with and without a psychiatric diagnosis were very well separated on the general factor (AUC 83 % [80, 87 %], *p* < 0.001, Δ = 1.7, *d* = 1.3), the optimal cut-off being θ = 0.68, approximately corresponding to a GSI of 0.72 (83 % specificity, 72 % sensitivity, DOR 12.5). ROC curves are shown in Fig. 3.

## Discussion

In this study we analyzed the psychometric properties of the SCL-90 questionnaire in adolescent inpatients and a community sample. We found the measurement invariance to be satisfactory between patient and control responses and between patients at admission and discharge. We also examined the dimensionality of measurement with methods intended for exploratory factor analysis and via confirmatory factor and bifactor analysis. The explained common variance was estimated for the latter. To better understand the viability of subscales, we also calculated omega-hierarchical and omega-subscale indices. Receiver operating curves were calculated in order to evaluate the SCL-90s ability to distinguish between controls and patients and between individuals with and without a psychiatric diagnosis.

Measurement invariance analyses revealed sufficient measurement invariance across patients and controls and across time points, in line with an earlier clinical and general population study of adults [51]. These findings support using all the items for the GSI or a general factor, though at least one but perhaps a few items show enough DIF in the unidimensional model to be considered for exclusion. The sample sizes were unfortunately too small to formally test structural invariance in multidimensional models.

We calculated estimates of the number of empirically found number of dimensions, which were highly divergent, and therefore limited our factor analyses to confirmatory testing of previously proposed models. The fit of the unidimensional factor model proved adequate, but the nine-factor structure of the SCL-90 proposed by the original author of the scale [8] was not supported, as it showed poor fit and very highly correlated subscales. In contrast, the bifactor model with one general factor of symptoms and the same nine specific factors yielded an excellent fit to the data in all three subsamples (patient admission, patient discharge, and controls). Similar results have been found also by Urbán et al. [29] and Thomas [52].

As in the previous study by Urbán et al. [29] with an adult sample, we observed a strong global distress factor and weaker specific symptom factors in our patient sample, while our control sample data appeared unidimensional. There are some other previous studies that have similar results among adults. For example, Paap et al. [53, 54] have also found that different populations have varying dimensionality results using Mokken scale analysis: while samples of patients with high levels of distress support multidimensionality of the SCL-90 [53], samples characterized by a low level of distress indicate unidimensionality [54]. Lastly, adolescent inpatients usually suffer from comorbid disorders, and symptomatically homogenous groups without symptoms of other mental disorders are rarely found [55], which may explain the strong unidimensionality also in our clinical sample.

The explained common variance (ECV) index reflected the same findings on dimensionality and higher level of distress in our study. In our patient admission subsample, with severe distress, the ECV of the general factor was 56 %, which means that the explained variance is approximately equally spread across general and group factors, while at discharge, the common variance explained by the general factor was 76 %, and the highest ECV was found in the control sample 82 %, which approaches unidimensionality [26]. Interestingly, in the study by Urbán et al. [29] their adult community sample had almost the same ECV index (83 %) as our adolescent controls, which implies continuity across age groups for this measurement property.

Overall, the analysis of general- and domain-specific components yielded strong support for the presence of a general factor of symptoms within the SCL-90 items and, on the other hand, gave limited evidence for the viability of the a priori multidimensional structure even in the inpatient admission sample. The specific symptom factors Phobic Anxiety (ω_s_ = 0.40) and Hostility (ω_s_ = 0.32) had the strongest, but still weak, contributions to explaining the variance of the admission responses. These same two subscales had the strongest coefficients also in the patient discharge and control samples. These two factors also stood out in the study by Urbán et al. [29], indicating that these subfactors are more independent or distinct from other subscales of the SCL-90. The weakest reliability coefficients in this study was found for the depression subscale, suggesting that the depression items in the SCL-90 measure general distress addressed by the whole questionnaire, and that the depression scale does not reflect depression specific factor of symptoms. Thus, the nine subscales demonstrated low reliability as estimated by omega subscale coefficients, showing that these subscales comprise too small amount of reliable variance to reliably interpret. The results of the present research suggest that there is limited value in using the very highly correlated SCL-90 subscale scores among adolescents, because they primarily reflect variations in general symptoms.

Summed raw scores correlated extremely well with scores on the general factor, which is expected with a large number of items and a strong general factor, and the association was stable across the score range. Sum scores can thus confidently be used as a proxy for the latent factor. In this study factor score distributions discriminated well between patient at admission, patients at discharge, and controls. The scores of the patient admission sample were clearly higher than the scores of the patient discharge, being lowest in the controls. Our community sample seemed to exhibit somewhat lower SCL-90 GSI scores than those of an Italian community sample of 15- to 19-year-old adolescents [24]. However, the profile of our sample and that of a previous Swedish community sample of adolescents under 20 years of age [13] resembled each other, showing that there may be some cultural differences in the proneness to report symptoms.

The SCL-90s screening properties as investigated with ROC analyses indicated that it adequately discriminates patients from the community sample and individuals with psychiatric diagnosis from those without, a result resembling those of earlier studies among adult patients [6, 10]. Adequate discrimination was found also between patients at admission, who have severe symptoms, and the same patients at discharge who were largely recovered but still symptomatic. This finding supports earlier studies [19] that the SCL-90 is also a sensitive tool to measure changes in symptoms. Interestingly, the overall information yielded by the questionnaire was highest at discharge, perhaps reflecting an improved ability to understand the items.

### Strengths and limitations

Strengths of this study include a relatively high number of consecutive inpatients and a sample of community youth matched for age and gender. Almost identical study protocols were used in both groups, and patients were followed prospectively. Furthermore, the psychiatric diagnoses were based on highly reliable and valid K-SADS-PL interviews, supplemented by patient records. The SCL-90 is a widely used and established questionnaire in clinical practice. A limitation of our study is the relatively small participation rate in the comparison group. A partial explanation is that participants had to have written informed consent from their legal guardians, and refusals were thus not necessarily due to the approached individual’s preferences. In addition, participants were asked to take part in a five-year follow-up study, and in this context, give their permissions for researchers to acquire information from official records concerning for example their future criminal records and use of health services.

These expectations may have influenced students’ willingness to participate in the study. However, we ascertained that community sample participants and non-participants did not differ on a number of socioeconomic variables used in matching, showing that our sampling was representative in this respect. The overall sample size was also too small for testing the measurement invariance of multifactorial models.

## Conclusions and clinical implications

As the confirmatory bifactor model improved on the unidimensional model in all subsamples on all fit indices, and achieved excellent fit, it can be considered a sufficient description of the data. As most subscales had a very small contribution, however, it would be interesting to perform exploratory bifactor analyses in future studies. Nevertheless, the general factor was dominant, and the SCL-90 can thus be used as a unidimensional index of psychiatric distress, also when using the raw item score average (GSI). As the subscales were poorly distinguishable from the main factor and each other, they should be considered to measure mostly general distress, and their use to assess separate symptom dimensions does not appear warranted. Among adolescents, the SCL-90 appears to be a useful screening tool as well as a valuable instrument for assessing change in average symptom levels within patient populations.

## Abbreviations

AUC: area under the curve
CFA: confirmatory factor analysis
CFI: comparative fit index
DIF: differential item functioning
DOR: diagnostic odds ratios
ECV: explained common variance index
GSI: global severity index
K-SADS-PL: the Schedule for Affective Disorders and Schizophrenia for School-Age Children—Present and Lifetime
MAP: minimum average partial correlation
ML: maximum likelihood
PA: parallel analysis
RMSEA: root mean square error of approximation
ROC: receiver operating characteristic
SCL-90: the Symptom Checklist-90
VSS: very simple structure
WLS: weighted least-squares
WLSMV: weighted least squares mean and variance adjusted
WRMR: weighted root mean square residual
